# A Brief Cognitive Behavioral Therapy–Based Digital Intervention for Reducing Hazardous Alcohol Use in South Korea: Development and Prospective Pilot Study

**DOI:** 10.2196/64459

**Published:** 2025-03-19

**Authors:** Manjae Kwon, Daa Un Moon, Minjae Kang, Young-Chul Jung

**Affiliations:** 1 Department of Psychiatry, Yonsei University College of Medicine, Seoul, Republic of Korea; 2 Institute of Behavioral Science in Medicine, Yonsei University College of Medicine, Seoul, Republic of Korea; 3 Department of Psychiatry and Neurosciences, Charité Campus Mitte, Charité Universitätsmedizin Berlin, Corporate Member of Freie Universität and Humboldt-Universität zu Berlin, Berlin, Germany

**Keywords:** alcohol, hazardous alcohol use, digital intervention, cognitive behavioral therapy, mobile apps, prevention, therapy-based, cognitive behavioral, alcohol use, South Korea, prospective pilot study, pilot study, alcohol consumption, death, disability, chronic medical condition, digital health interventions, traditional treatment methods, Korean, hazardous drinking, acceptability, feasibility, smartphone app, alcohol use disorder, psychiatric symptoms, mobile phone

## Abstract

**Background:**

Alcohol consumption is a leading cause of death and disability worldwide, associated with numerous acute and chronic medical conditions. Digital health interventions offer a promising solution to overcome barriers associated with traditional treatment methods, providing accessible, scalable, and cost-effective means to support individuals in reducing hazardous drinking.

**Objective:**

This pilot study aims to evaluate the feasibility, acceptability, and preliminary efficacy of the Sober smartphone app in individuals with hazardous alcohol use.

**Methods:**

This single-group, pre- and postpilot study included 20 participants with risky alcohol use, identified using the Alcohol Use Disorder Identification Test. Participants used the Sober app for 4 weeks, incorporating cognitive behavioral therapy–based interventions. Feasibility was assessed by study and session completion rates, acceptability by participant satisfaction and perceived usefulness, and preliminary efficacy by changes in alcohol consumption and psychiatric symptoms. Semistructured interviews with participants and clinicians provided qualitative perspectives on the app’s usability, efficacy, and areas for improvement.

**Results:**

Of the 20 enrolled participants, 17 completed the study. The app demonstrated high feasibility with an 85% (17/20) study completion rate, and 59% (10/17) completed all cognitive behavioral therapy sessions. Participants reported positive acceptability, with average satisfaction and usefulness ratings of 3.8 and 3.7 of 5, respectively. Preliminary efficacy outcomes showed significant improvements: abstinence days increased from 67% to 85% (*z*=−3.17; *P*=.002), heavy drinking episodes decreased from 3.3 to 1.9 (*t*_16_=−2.97; *P*=.003), and total alcohol consumption reduced from 456.8 to 195.9 mL (*t*_16_=3.16; *P*=.002). Alcohol Use Disorder Identification Test scores dropped from 17.5 to 10.7 (*t*_16_=4.51; *P*<.001). Additionally, depression (Patient Health Questionnaire-9) scores decreased from 5.8 to 4.4 (*t*_16_=2.91; *P*=.01), and anxiety (Generalized Anxiety Disorder-7) scores from 3.4 to 2.1 (*z*=−2.80; *P=*.005). No adverse events were reported. Qualitative analysis found participants valued daily logging but noted usability issues, while clinicians called for tailored goals, enhanced communication features, and age-specific content.

**Conclusions:**

The mobile app Sober shows promise as an effective tool for reducing hazardous alcohol consumption and improving related psychiatric symptoms. The study demonstrated high feasibility and positive acceptability, with significant preliminary efficacy in reducing alcohol use. Qualitative findings provided actionable evidence for refining the app’s usability and clinical integration. Further research through a randomized controlled trial is warranted to confirm these findings and optimize the app’s features and content.

## Introduction

Alcohol is the most widely used psychoactive substance worldwide and poses a significant public health problem with a massive socioeconomic burden [1,2]. It is a leading cause of death and disability globally, contributing to a wide range of acute and chronic medical conditions. Acute medical consequences of high alcohol intake include injuries, car accidents, and violence [3]. Chronic diseases associated with alcohol consumption include liver cirrhosis, cardiovascular diseases, and various cancers [4-8]. Additionally, alcohol use disorders are linked to numerous psychiatric comorbidities, such as mood disorders, anxiety disorders, personality disorders, and suicide [9,10].

Hazardous drinking, defined as a drinking behavior that significantly increases the risk of adverse alcohol-related outcomes [11], is prevalent among those who consume alcohol, with a global prevalence of 17% and 15.1% in South Korea [2,12]. Effective prevention typically includes screening and brief interventions to identify and address hazardous drinking early [13-15]. These interventions involve trained staff using a brief screening tool combined with motivational interviewing techniques to provide personalized feedback and clinical options tailored to the individual’s level of risk, including a referral to treatment component, if necessary [11,16-18]. However, several barriers hinder the implementation of these interventions, including concerns about stigmatization, lack of resources, insufficient training, high clinician workload, and logistical and financial obstacles for patients seeking help [19-22].

Digital health interventions offer a promising solution to overcome this barrier, especially in countries like South Korea, which has the highest smartphone ownership rate in the world at approximately 95%, with minimal generational gaps in ownership [23]. With near-ubiquitous smartphone ownership, these interventions can improve health outcomes by providing inexpensive, easily accessible, and scalable methods to deliver education, support, and monitoring through personal devices [24]. Currently, many apps in commercial app stores aim to help users reduce their alcohol consumption, but few have been rigorously evaluated, and only a minority have published efficacy information [25]. While the overall evidence for alcohol reduction through digital interventions is promising, it remains inconclusive [25-31]. Moreover, reporting theoretical foundations in these apps is often limited and unclear, indicating a need for more rigorous evaluation and transparency in their development [31].

Hazardous alcohol use frequently co-occurs with psychological distress, including symptoms of depression and anxiety [32]. These symptoms often create a cyclical relationship with problematic alcohol use, where they exacerbate drinking patterns and complicate treatment outcomes [33,34]. Research shows that brief interventions targeting hazardous alcohol use can reduce consumption and alleviate psychological distress, depression, and anxiety, addressing both alcohol use and co-occurring mental health symptoms [35,36]. Studies also highlight the potential of digital interventions to achieve similar dual benefits, providing scalable solutions to this complex challenge [37,38].

This pilot study aims to estimate the potential of a digital intervention using the Sober smartphone app (WELT Corp Ltd) for secondary prevention in individuals with hazardous alcohol use. The primary objective is to evaluate the app’s feasibility, acceptability, and preliminary efficacy in reducing hazardous drinking behaviors. In addition, the study examines secondary outcomes, including the impact of the intervention on symptoms such as depression, anxiety, and stress. The findings from this pilot study will be instrumental in refining the app’s features and content and optimizing the study design for a planned randomized controlled trial (RCT).

## Methods

### Design and Setting

This single-group, pre- and postpilot study assessed the preliminary efficacy, feasibility, and acceptability of the mobile app Sober for reducing hazardous alcohol consumption. The single-group design was chosen to provide initial insights into the intervention’s effects and to inform the design of future RCTs.

### Ethical Considerations

The study was approved by the institutional review board of Yonsei Severance Hospital (approval 4-2023-0338) and adhered to the ethical standards of the Declaration of Helsinki. Prior to study participation, all participants provided written informed consent, including consent for the collection and analysis of their data. Participants were informed of their right to withdraw from the study at any time. No modifications to the content or methodology were made after the study commenced. We anonymized the participants using unique ID numbers. No identifying or potentially identifying information is included in this paper or MultimediaAppendices 1  2. All identifying or potentially identifying data are securely maintained by Yonsei Severance Hospital and accessible only to the authorized research team. The participants received approximately US $34 for each clinic visit for their time and travel expenses.

### Recruitment

Participants were recruited through outpatient clinic bulletin boards, web-based advertisements (eg, university websites), flyers, and word of mouth. Recruitment targeted individuals who self-identified as having concerns about their drinking habits and sought mental health support. The study was conducted at Yonsei Severance Hospital, a tertiary care teaching hospital in Seoul, South Korea. Interested individuals received detailed information about the study and underwent an in-person screening process after providing written informed consent. Board-certified psychiatrists (MK, DUM, and YCJ) reviewed each participant’s medical and medication history to determine eligibility following a psychiatric assessment [39], including a review of medical and medication history.

### Eligibility Criteria

Participants aged 19‐65 years who scored 8 or higher on the Alcohol Use Disorder Identification Test (AUDIT) [40,41] were included, with risky alcohol use encompassing hazardous use, harmful use, and probable dependence. Additional inclusion criteria required participants to possess sufficient Korean language skills, own an Android smartphone, and have no difficulty using mobile apps independently. They also needed to understand the study information and provide written informed consent. Exclusion criteria included major psychiatric disorders such as schizophrenia spectrum disorders, bipolar disorder, and major depressive disorder. Participants with substance use disorders involving substances other than alcohol or nicotine (eg, illicit drugs or prescription medications) were excluded. Participants with active and progressive physical illnesses, unstable medical conditions, or a life expectancy of less than 6 months were also excluded. Pregnant individuals or those planning to become pregnant during the study period, as well as individuals who participated in another clinical trial within 4 weeks before screening, were not eligible.

### Procedure

Enrollment and follow-up for the study occurred between May and August 2023 (Table 1). Participants who consented to join the pilot study downloaded the Sober app and were given usernames to sign in. All participants were encouraged to use the app for self-monitoring and to log in regularly over the 4 weeks. Additionally, participants were provided with a Samsung Galaxy Smart Watch 5 (model: SM-R900NZAAKOO) to collect biometric data throughout the study. Participants were required to visit the clinic 3 times: at baseline (visit 1), 2 weeks after the baseline visit (visit 2), and 4 weeks after the baseline visit (visit 3) for assessments. Each visit included a consultation with a board-certified psychiatrist who provided standard psychiatric consultation regarding alcohol consumption based on the data entered into the mobile app.

**Table 1.** Schedule of enrollment, interventions, and assessments for a single-group, pre-post pilot study evaluating the feasibility, acceptability, and preliminary efficacy of the Sober mobile app^a^.

	Study period and time points			
	Enrollment	Visit 1	Visit 2	Visit 3 and closeout
	0 days	0 days	14±4 days	28±4 days
**Enrollment**				
Eligibility screen and informed consent	✓			
**Intervention**				
Mobile app Sober and consultation with a psychiatrist at each visit		✓	✓	✓
**Assessments**				
Sociodemographic and clinical characteristics	✓			
Alcohol consumption history	✓			
**Primary objectives**				
Feasibility of the Sober app			✓	✓
Acceptability of the Sober app				✓
Days abstinent in the past 2 weeks (%)		✓	✓	✓
**Secondary objectives**				
AUDIT^b^	✓		✓	✓
PHQ-9^c^		✓	✓	✓
GAD-7^d^		✓	✓	✓
PSS^e^		✓	✓	✓
CIWA-Ar^f^		✓	✓	✓
Amount of alcohol consumed (mL) in the past 2 weeks		✓	✓	✓
Adverse events		✓	✓	✓

a The study was conducted at Yonsei Severance Hospital, Seoul, South Korea, from May to August 2023.

b AUDIT: Alcohol Use Disorders Identification Test.

c PHQ-9: Patient Health Questionnaire-9.

d GAD-7: Generalized Anxiety Disorder-7.

e PSS: Perceived Stress Scale.

f CIWA-Ar: Clinical Institute Withdrawal Assessment of Alcohol Scale—Revised.

### Sober App

**Figure 1.**
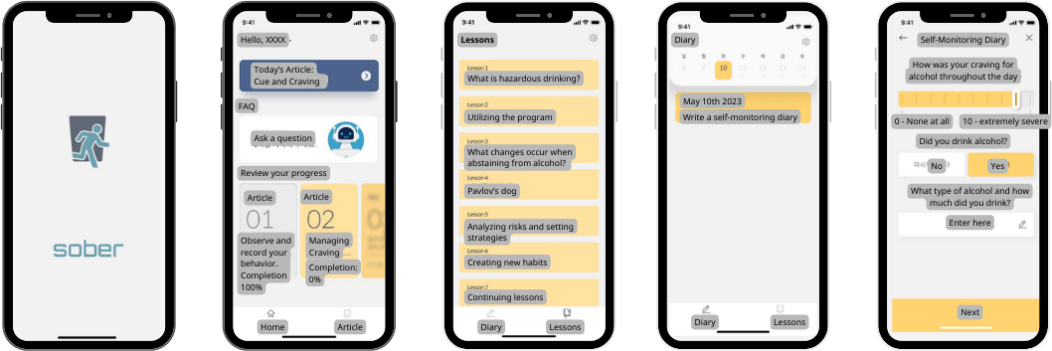
Screenshots of the Sober app’s interface. Illustrative screenshots of the mobile app used in this study, showcasing features for daily logging, educational modules, and progress tracking. Original content in Korean has been translated into English for presentation.

The Sober app was developed as a digital intervention tool to reduce and prevent hazardous drinking (Figure 1). The development process involved a multidisciplinary team of experienced clinicians, software developers, product designers, and behavioral scientists to ensure clinical effectiveness and user-friendliness. Before developing the program, the team reviewed relevant materials, such as treatment manuals, intervention descriptions, guidelines, patient reports, and trial results. The program’s development involved iterative evaluations of prototypes, with the findings used to refine and enhance the final app design. Designed for Android smartphones, the app supports clinicians, especially those with minimal addiction training, in providing structured, evidence-based interventions to hazardous drinkers in the general population. The cognitive behavioral therapy (CBT)–based app incorporates interventions that include psychoeducation, motivational interviewing, cognitive behavioral skills training (eg, problem-solving and behavior substitution), and personalized normative feedback. It provides content on the effects of alcohol on the brain and behavior, risk awareness, and strategies for reducing risky drinking habits while encouraging users to consider alternatives to heavy drinking.

The modularly structured app features educational modules, self-monitoring tools, and interactive elements. The CBT-based modules are divided into 4 chapters, which include 8 sessions, each addressing different aspects of hazardous drinking and providing skills training and risk awareness (Table 2). The content is organized sequentially. The first chapter, “What is hazardous drinking?,” introduces participants to the concept and helps them set initial goals, such as abstaining from alcohol for 4 weeks, entering self-monitoring reports, and engaging in the CBT program. Participants also receive personalized normative feedback on their drinking habits. The second chapter, “What changes occur when abstaining from alcohol?,” covers psychoeducation about changes in sleep, appetite, and emotions resulting from abstinence, with participants recording these changes in their self-monitoring logs. The third chapter, “Pavlov’s dog,” explains the concepts of cues, cravings, behaviors, and outcomes, helping participants identify high-risk situations and understand how cravings lead to drinking behavior. This chapter guides participants in exploring and selecting alternative behaviors to replace drinking. The fourth chapter, “Forming a healthy drinking habit,” reviews the previous 4 weeks and reinforces the skills learned. To facilitate self-monitoring and promote self-awareness, the app included a daily log function, where participants recorded their alcohol consumption, mood, appetite, and sleep quality.

**Table 2.** Description of cognitive behavioral therapy–based modules in the Sober mobile app intervention.

Week and chapter		Session	Key principles and contents
**Week 1**			
	What is hazardous drinking?	Do I belong to high-risk drinking group?	Introduction to the High-Risk Drinking Prevention Program: this includes a description of high-risk drinking behaviors and a self-assessment for participants to evaluate their own drinking habits. Normative feedback is provided for the participants’ level of current drinking habit. The program also offers psychoeducation about the long-term risks associated with continuing these drinking habits.
	What is hazardous drinking?	Promise for 4 weeks abstinence	We set up the action plan with the initial goal of abstaining from alcohol for the next 4 weeks while participating in the program. Evaluate individuals’ expectations regarding the outcomes of alcohol abstinence. Provide instructions for recording sleep, emotions, appetite, and alcohol consumption in the app.
**Week 2**			
	What changes occur when abstaining from alcohol?	Alcohol and sleep	Psychoeducation regarding the link between alcohol and sleep.
	What changes occur when abstaining from alcohol?	Alcohol and appetite	Psychoeducation regarding the link between alcohol and appetite.
	What changes occur when abstaining from alcohol?	Alcohol and emotion	Psychoeducation regarding the link between alcohol and emotion.
**Week 3**			
	Pavlov’s dog	Cue	Description of the cue-craving-behavior-result cycle in alcohol consumption. This session focuses on identifying the cues that lead to this cycle.
	Pavlov’s dog	Craving	Psychoeducation on the various ways “craving” may be identified in participants. Participants will set up alternative behaviors to resort to when they experience alcohol cravings.
**Week 4**			
	Forming a healthy drinking habit	Review and relapse prevention	Review of the intervention and establishment of relapse prevention strategies.

### Assessments

At baseline (visit 1), participants provided sociodemographic information, lifestyle habits, past disease history, previous medication, and detailed information on their alcohol consumption habits. At each of the 3 visits, participants were evaluated using several standardized instruments: The AUDIT was used to assess hazardous and harmful patterns of alcohol consumption [40,41]. Symptoms of depression were measured using the Patient Health Questionnaire-9 (PHQ-9) [42,43], while symptoms of anxiety were evaluated using the Generalized Anxiety Disorder-7 (GAD-7) scale [44,45]. The Perceived Stress Scale (PSS) was used to assess perceived levels of stress [46,47], and the Clinical Institute Withdrawal Assessment of Alcohol Scale—Revised (CIWA-Ar) was used to evaluate alcohol withdrawal symptoms [48,49]. At the final visit (visit 3), the completion rate of the app-based sessions was assessed along with participants’ satisfaction ratings, perceived usefulness of the app, and ease of mastering the app program, all assessed through a 5-point Likert scale. Adverse events were monitored and recorded at each visit to ensure participant safety.

### Outcomes

The study’s primary outcomes focused on feasibility, acceptability, and preliminary efficacy. Feasibility was assessed at visit 3 based on the study completion rate and the completion of all 8 CBT-based intervention sessions. Acceptability was evaluated at visit 3 using 3 measures: participant satisfaction, perceived usefulness of the app, and ease of mastering the app.

Preliminary efficacy was analyzed by comparing outcomes between baseline (visit 1) and postintervention (visit 3). The primary outcome was the change in the number of days participants abstained from alcohol in the past 14 days. Secondary outcomes included changes in total AUDIT scores, the total amount of alcohol consumed in the past 14 days, and psychiatric symptom scores (PHQ-9, GAD-7, PSS, and CIWA-Ar), all assessed at both baseline (visit 1) and postintervention (visit 3).

### Statistical Analysis

All statistical analyses were conducted using SPSS (version 26 for Windows; IBM Corp). Frequencies and percentages described categorical variables, while means and SDs were used for continuous variables. Depending on the data distribution’s normality (Shapiro-Wilk test), paired *t* tests (2-tailed) or Wilcoxon signed rank tests were used to compare pre- and postintervention outcomes. Statistical significance was set at *P*<.05. Results were reported with corresponding *P* values.

### Participant and Clinician Interviews

Qualitative data were collected through semistructured, open-ended interviews conducted at the final visit (visit 3). All 17 participants who completed the study participated in individual interviews to explore their experiences with the app and the clinical trial. Additionally, 2 clinicians involved in the trial were interviewed separately following the study’s conclusion to provide insights from a clinical perspective.

The interviews were conducted face-to-face (in private rooms at the study site) or via videoconferencing, depending on participant preferences, and lasted approximately 20‐30 minutes. An interview guide was used to ensure consistency across interviews. For participants, questions focused on app usability, content relevance, and perceived impact on drinking behaviors. For clinicians, the interviews explored the app’s integration into clinical workflows, feasibility, and suggestions for tailoring the intervention to diverse patient needs. All interviews were conducted by members of the research team involved in the study. Interviews were audio-recorded with participant consent, transcribed verbatim, and anonymized to maintain confidentiality.

Thematic analysis was used to analyze the qualitative data, following Braun and Clarke’s 6-phase framework [50]: 2 independent researchers (DUM and Yujin Lee) coded the transcripts to identify key patterns and themes. Codes were grouped into overarching themes and refined iteratively to ensure they accurately represented the data. Representative verbatim quotes were selected to illustrate key findings and provide depth to the analysis. Discrepancies in coding were resolved through discussion. The findings are reported following the COREQ (Consolidated Criteria for Reporting Qualitative Research) guidelines [51].

## Results

### Participant Eligibility and Baseline Characteristics

**Figure 2.**
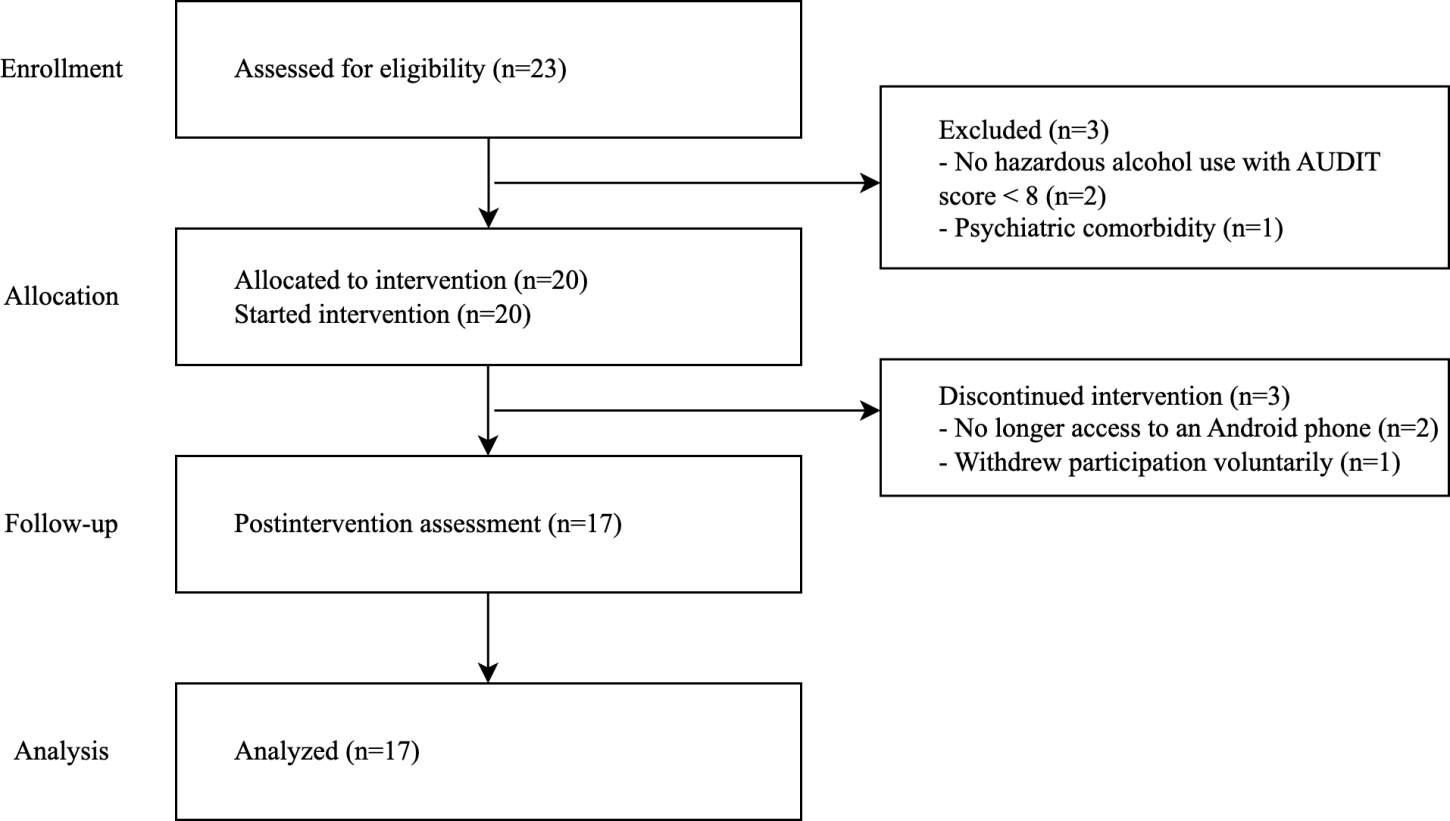
Flowchart summarizing the participant recruitment, screening, enrollment, intervention, and follow-up processes. AUDIT: Alcohol Use Disorders Identification Test.

A total of 20 eligible participants with hazardous alcohol use, identified based on their AUDIT scores, were enrolled in the study. Of these, 17 completed the 4-week clinical trial. In total, 2 participants dropped out because they no longer had access to Android smartphones, and 1 withdrew voluntarily (Figure 2). All participants were male, with a mean age of 22.29 (SD 8.3) years. Most were students and single. Regarding smoking status, 3 participants were current smokers, 3 were ex-smokers, and 11 were never smokers. The mean age at first alcohol drinking was 18.9 (SD 1.2) years, and the mean age at first heavy drinking episode was 19.7 (SD 1.0) years. In total, 7 participants had a family history of alcohol-related problems, and 3 showed insights into their alcohol problems (Table 3).

**Table 3.** Participants’ baseline characteristics (n=17).

Characteristics	Values
Sex (male), n (%)	17 (100)
Age (years), mean (SD)	22.29 (8.3)
Height (cm), mean (SD)	174.9 (3.9)
Weight (kg), mean (SD)	74.4 (12.8)
**Education, n (%)**	
Middle school	1 (6)
High school	12 (71)
Bachelor’s degree	3 (18)
Master’s degree or postgraduate	1 (6)
**Occupation, n (%)**	
Student	14 (82)
Office worker	1 (6)
Labor worker	1 (6)
Unemployed	1 (6)
**Religion, n (%)**	
Christian	1 (6)
Others	0 (0)
None	16 (94)
**Marital status, n (%)**	
Single	16 (94)
Married	0 (0)
Divorced	1 (6)
**Living arrangement, n (%)**	
Living alone	8 (47)
Living with others	9 (53)
**Smoking status, n (%)**	
Current smokers	3 (18)
Ex-smoker	3 (18)
Never smoker	11 (65)
Age at first alcohol drinking (years), mean (SD)	18.9 (1.2)
Age at first binge drinking (years), mean (SD)	19.7 (1.0)
**Presence of alcohol-related accident, n (%)**	
No	10 (59)
Yes (once)	5 (29)
Yes (multiple)	2 (12)
**Presence of alcohol-related legal problems, n (%)**	
No	16 (94)
Yes (once)	0 (0)
Yes (multiple)	1 (56)
**Presence of drunken driving, n (%)**	
No	17 (100)
Yes	0 (0)
**Family history of alcohol-related problems, n (%)**	
No	10 (59)
Yes	7 (41)
**Insight of alcohol problems, n (%)**	
No	14 (82)
Yes	3 (18)

### Feasibility and Acceptability

The feasibility of the intervention was assessed based on the completion rates of the study and the CBT-based educational sessions within the app. Of the 20 participants initially enrolled, 17 (85%) completed the study. Among these 17 participants, 10 (59%) completed all 8 CBT-based sessions. Acceptability was evaluated in the 17 participants who completed the final visit (visit 3). Participants were asked to rate their satisfaction with the app on a scale from 0=not satisfied at all to 5=very satisfied. The average satisfaction score was 3.8 (SD 0.9). When asked to rate the usefulness of the app from 0=not useful at all to 5=very useful, the average response was 3.7 (SD 0.9). Additionally, participants rated their ease of mastering the app on a scale from 0=could not use without help to 5=used naturally without difficulty, with an average score of 3.5 (SD 0.6).

### Preliminary Efficacy Outcomes

Preliminary efficacy outcomes showed significant improvements in several key measures (Table 4). The percentage of days participants remained abstinent increased from 67% to 85% (*z*=−3.17; *P*=.002). The frequency of heavy drinking episodes decreased from 3.3 to 1.9 (*t*_16_=−2.97; *P*=.003). The amount of alcohol consumed reduced significantly from 456.8 to 195.9 mL (*t*_16_=3.16; *P*=.002). Total AUDIT scores dropped from 17.5 to 10.7 (*t*_16_=4.51; *P*<.001). Regarding psychological assessment outcomes, PHQ-9 scores decreased from 5.8 to 4.4 (*t*_16_=2.91; *P*=.01), and GAD-7 scores from 3.4 to 2.1 (*z*=−2.80; *P*=.005). There were nonsignificant changes in PSS scores (17.2 to 16.1; *P*=.19) and CIWA-Ar scores (0.3 to 0.8; *P*=.74). No adverse events were reported by the study participants.

**Table 4.** Changes in alcohol consumption, psychological symptoms, and related measures before and after a 4-week intervention using the mobile app “Sober.”

	Baseline (version 1), mean (SD)	Postintervention (version 3), mean (SD)	Difference, mean (SD)	*t* test (*df*=16)^a^	*P* value
Days abstinent in the past 2 weeks (%)	67 (25)	85 (16)	18.0 (4.2)	*z*=−3.17^b^	.002
Frequency of heavy drinking (AUDIT^c^, question 3)	3.3 (0.7)	1.9 (1.4)	−1.4 (0.3)	−2.97	.003
Amount of alcohol consumed (mL)	456.8 (441.9)	195.9 (320.2)	−260.9 (82.6)	3.16	.002
AUDIT	17.5 (6.2)	10.7 (9.0)	−6.7 (1.5)	4.51	<.001
PHQ-9^d^	5.8 (5.4)	4.4 (5.6)	−1.4 (0.5)	2.91	.01
GAD-7^e^	3.4 (3.4)	2.1 (3.5)	−1.3 (0.5)	*z*=−2.80^b^	.005
PSS^f^	17.2 (2.9)	16.1 (2.6)	−1.1 (0.8)	1.38	.19
CIWA-Ar^g^	0.3 (0.6)	0.8 (2.7)	0.5 (0.7)	*z*=0.01^b^	.74

a Paired *t* test (2-tailed).

b Wilcoxon signed rank test.

c AUDIT: Alcohol Use Disorder Identification Test.

d PHQ-9: Patient Health Questionnaire-9.

e GAD-7: Generalized Anxiety Disorder-7.

f PSS: Perceived Stress Scale.

g CIWA-Ar: Clinical Institute Withdrawal Assessment of Alcohol Scale—Revised.

### Participant and Clinician Perspectives on the Intervention

Qualitative feedback from participants and clinicians provided insights into the app’s usability, effectiveness, and areas for improvement. For a detailed summary, see MultimediaAppendices 1  2.

#### Participant Feedback

Interviews with the 17 participants highlighted 3 primary themes: behavioral impact, usability challenges, and content suggestions.

Participants consistently reported that using the app increased their awareness of drinking behaviors and encouraged self-reflection, which helped them reduce alcohol consumption:

When I logged my drinking every day, it made me stop and think about how much I was drinking. It naturally helped me reduce my alcohol consumption.

[Participant 1]

Tracking how often and how much I drink made me more aware of my habits.

[Participant 3]

Participants also noted the app’s role in helping them observe connections between their drinking and other aspects of their health, such as sleep and appetite:

It was helpful to see how my drinking affected things like my appetite and sleep. It made me think about how drinking impacts my health.

[Participant 13]

However, a participant reported unintended effects, such as increased cravings when focusing on abstinence:

Sometimes, thinking about not drinking actually made me want to drink more. It felt like a trigger at times.

[Participant 7]

Usability issues were commonly mentioned, including app crashes, lack of notifications, and vague criteria. Participants suggested improvements to enhance functionality:

The app would crash a lot when I tried to log something, which was really frustrating.

[Participant 6]

I wish the app had reminders, like notifications in the morning to remind me to log.

[Participant 4]

It felt like some of the criteria in the app were too vague. I wasn’t always sure what I was supposed to do.

[Participant 3]

It would be nice to see trends over time, like a graph showing my progress.

[Participant 9]

Participants also recommended expanding the app’s content with more educational materials, such as strategies for managing cravings and understanding the risks of alcohol consumption:

The app could include more about the long-term effects of drinking.

[Participant 17]

It would be great if I could log more details, like my emotions or cravings. Just using a simple scale didn’t capture everything.

[Participant 12]

#### Clinician Feedback

Interviews with 2 clinicians involved in the trial provided additional perspectives on the app’s clinical utility and suggestions for improvement.

Clinicians observed that participants who recorded their mood and sleep alongside abstinence became more aware of positive changes in their mental and physical health, reinforcing their motivation to reduce alcohol consumption:

By recording their mood and sleep daily while abstaining from alcohol, participants seemed to independently recognize the positive changes in their mind and body, which, in turn, reinforced their commitment to staying alcohol-free.

[Clinician 1]

They emphasized the importance of flexibility in accommodating participants’ diverse goals, such as achieving abstinence or reducing binge episodes, suggesting features like weekly goal setting and tracking achievements:

Allowing for participants to set personalized goals every week and adding features that allow participants to record their achievement would be beneficial.

[Clinician 1]

To enhance clinical integration, clinicians recommended features that facilitate communication and monitoring, such as messaging options and tools to assess participants’ motivation:

Features that connect clinicians and patients, like messaging or feedback options, would make follow-ups easier and more effective.

[Clinician 2]

If patients’ motivation levels for change can be assessed through the app, it will help clinicians effectively tailor their approach to patient interventions in real-life settings.

[Clinician 2]

Additionally, clinicians underscored the importance of tailoring app content to different age groups and suggested incorporating features that allow participants to reflect on their motivations for change:

Younger and older users have very different needs and expectations. The app should include content that’s tailored to different age groups.

[Clinician 2]

## Discussion

### Principal Findings

This pilot study aimed to assess the feasibility, acceptability, and preliminary efficacy of the Sober mobile app for reducing hazardous alcohol consumption. The results demonstrated significant improvements in key measures of alcohol use, including increased days of abstinence, reduction in heavy drinking episodes, and decreased overall alcohol consumption. Additionally, notable reductions in AUDIT scores and improvements in psychological assessments (PHQ-9 and GAD-7 scores) were observed.

Qualitative feedback from participants and clinicians provided valuable insights into the app’s strengths and areas for improvement. Participants emphasized the app’s ability to increase awareness of drinking behaviors and promote self-reflection, which supported behavior change. However, usability challenges, such as technical issues and limited options for personalization, were highlighted. Clinicians stressed the importance of tailoring the app to diverse user goals and incorporating features to facilitate clinical integration. These findings will inform the refinement of the app and future iterations of the study design.

### Comparison With Prior Work

Our findings align with previous research indicating the effectiveness of digital interventions in reducing alcohol consumption, particularly among the young male population [52], which mirrors the demographic of our study. Other studies have also shown positive effects in the general population, suggesting that such interventions could have broad applicability [29,31,53-55]. The promising use of CBT in digital formats is consistent with existing evidence that CBT delivered in clinical settings can reduce alcohol consumption [11,56,57]. Other digital interventions using CBT-based approaches have also demonstrated effectiveness in reducing alcohol consumption [58-60]. However, in the CBT framework, various interventions, including normative feedback, self-monitoring, psychoeducation, action planning, goal setting, problem-solving skills, and identifying or managing triggers and cravings, aim to strengthen “reflective” cognitive processes used to control behavior [25]. This approach diversity highlights the need for a more standardized framework for developing and evaluating digital interventions [31]. This study also supports findings that digital interventions can positively impact co-occurring psychological symptoms, such as depression and anxiety, further emphasizing their dual utility [61,62].

The app’s high feasibility, demonstrated by an 85% (17/20) study completion rate, suggests that it is well-received and manageable for participants. However, the fact that only 59% (10/17) completed all 8 CBT-based educational sessions highlights the challenge of maintaining engagement with the full content. Strategies to enhance user engagement, such as incorporating gamification elements, personalized feedback, and real-time adjustments using predictive modeling [63-65], could address this gap.

Acceptability ratings were positive, with satisfaction and perceived usefulness ratings averaging 3.8 and 3.7 of 5, respectively. Feedback indicated that daily logging and self-monitoring features were valuable in promoting self-reflection and awareness of drinking habits. However, several usability improvements were suggested, such as showing averages of past drinking, fixing app crashes, adding notification settings, and integrating with other health apps. Clinician feedback emphasized the need for customizable goals, treatment periods, and content tailored to different age groups. These suggestions will be considered in future iterations of the app to ensure that it meets the diverse needs of its users. Furthermore, incorporating interactive elements and practical synthesis in workflows could enhance the overall intervention.

### Strengths and Limitations

This study demonstrated the potential of the Sober app for reducing risky alcohol consumption; yet, several limitations must be acknowledged. The single-arm, pre- and postdesign limits causal inferences, and the study was not sufficiently powered. The short duration of the study and lack of long-term follow-up limit the ability to assess the sustained effects of the intervention. The inclusion of face-to-face sessions with clinicians may have influenced the outcomes, as the reinforcement and availability of clinicians could have impacted the results [66]. Furthermore, the homogeneous participant demographic, consisting solely of young male participants, limits the generalizability of the findings.

Despite these limitations, the study had several notable strengths. To our knowledge, this is the first study to evaluate a brief digital intervention for hazardous alcohol use in South Korea. The significant preliminary efficacy demonstrated suggests potential for real-life clinical implementation. The high overall completion rate and positive acceptability ratings indicate that the app is well-received and safe to use. Importantly, the inclusion of qualitative feedback provides a nuanced understanding of user experiences, directly informing app improvements and study design.

At a population level, addressing interventions for individuals who exhibit hazardous drinking can have the greatest impact on reducing alcohol-related problems [67]. By providing an easily accessible, scalable, and cost-effective intervention, the Sober app could help reduce the overall burden of hazardous drinking on health care systems. While numerous studies have shown that screening and brief intervention can lead to significant decreases in alcohol use in primary care populations [13,68], digital intervention lessens the burden on primary care providers, requires no extensive training, and mitigates the fear of stigma, as interventions can be anonymous [69]. This is particularly relevant in South Korea, where hazardous drinking rates are significant [12], and smartphone ownership is nearly ubiquitous [23].

### Future Research

Future research should include an RCT to assess the app’s efficacy compared to standard care without app use, with larger samples and follow-up assessments to determine long-term effects. Expanding the study to include a more diverse population will enhance the generalizability of the findings [70]. Optimizing the app’s tailoring, structure, and content based on participant and clinician feedback is essential. Personalized content, interactive elements [71], gamification [72,73], and predictive modeling using data from daily logs and wearables could improve user engagement and intervention effectiveness by providing personalized feedback and real-time adjustments [63-65]. Integrating the app with other health monitoring tools and clinician workflow could give a more holistic approach to managing alcohol consumption [71,74].

### Conclusions

The mobile app Sober shows promise as an effective tool for reducing hazardous alcohol consumption and improving related psychiatric symptoms. This pilot study demonstrated significant preliminary efficacy, high overall feasibility, and positive acceptability. The app’s features, such as daily logging and CBT-based content, helped users develop greater self-awareness and control over their drinking habits, showing potential for integration into clinical practice as a supportive tool. Qualitative feedback from participants and clinicians identified critical areas for improvement, such as usability, goal customization, and age-specific content, which will guide future app refinement. With further research and refinement, the Sober app has the potential to make an impact on public health by providing a scalable and accessible intervention for hazardous drinking.

## Supplementary material

10.2196/64459 Multimedia Appendix 1 Participant codes with acceptability measures.

10.2196/64459 Multimedia Appendix 2 Thematic quotes from participant and clinician interviews.

## Abbreviations

AUDIT: Alcohol Use Disorders Identification Test
CBT: cognitive behavioral therapy
CIWA-Ar: Clinical Institute Withdrawal Assessment of Alcohol Scale—Revised
COREQ: Consolidated Criteria for Reporting Qualitative Research
GAD-7: Generalized Anxiety Disorder-7
PHQ-9: Patient Health Questionnaire-9
PSS: Perceived Stress Scale
RCT: randomized controlled trial
