# Supplementary material for: A Brief Cognitive Behavioral Therapy–Based Digital Intervention for Reducing Hazardous Alcohol Use in South Korea: Development and Prospective Pilot Study
Source: JMIR Form Res. 2025 Mar 19;9:e64459. doi: 10.2196/64459 (PMC11941278; doi:10.2196/64459)
Supplement: Multimedia Appendix 1 [file formative-v9-e64459-s001.docx]

| **Study participant codes** | **Satisfaction (1-5)** | **Perceived usefulness**  **(1-5)** | **Ease of mastering app**  **(1-5)** |
| --- | --- | --- | --- |
| P1 | 4 | 4 | 4 |
| P2 | 4 | 2 | 4 |
| P3 | 4 | 3 | 3 |
| P4 | 4 | 4 | 5 |
| P5 | 4 | 3 | 3 |
| P6 | 5 | 4 | 4 |
| P7 | 4 | 3 | 4 |
| P8 | 4 | 3 | 5 |
| P9 | 1 | 4 | 2 |
| P10 | 4 | 4 | 5 |
| P11 | 3 | 4 | 3 |
| P12 | 4 | 3 | 4 |
| P13 | 4 | 4 | 4 |
| P14 | 4 | 4 | 5 |
| P15 | 2 | 4 | 2 |
| P16 | 4 | 3 | 4 |
| P17 | 4 | 4 | 4 |

All measures were assessed on a 5-point Likert scale, where 1 = Very Dissatisfied/Not Useful/Very Difficult and 5 = Very Satisfied/Very Useful/Very Easy.
