# Supplementary material for: A Brief Cognitive Behavioral Therapy–Based Digital Intervention for Reducing Hazardous Alcohol Use in South Korea: Development and Prospective Pilot Study
Source: JMIR Form Res. 2025 Mar 19;9:e64459. doi: 10.2196/64459 (PMC11941278; doi:10.2196/64459)
Supplement: Multimedia Appendix 2 [file formative-v9-e64459-s002.docx]

| **Source** | **Theme** | **Quotes** |
| --- | --- | --- |
| **Participants** | Behavioral Impact | “When I logged my drinking every day, it made me stop and think about how much I was drinking. It naturally helped me reduce my alcohol consumption.” (P1)  “Tracking how often and how much I drink made me more aware of my habits” (P3)  “Awareness helped avoid or control drinking during situations or urges.” (P10)  “It was helpful to see how my drinking affected things like my appetite and sleep. It made me think about how drinking impacts my health.” (P13)  “Being aware of drinking was helpful.” (P14)  “Sometimes, thinking about not drinking actually made me want to drink more. It felt like a trigger at times.” (P7) |
|  | Usability | “The app would crash a lot when I tried to log something, which was really frustrating.” (P6)  “It would be better if the app presented drinking days more clearly on a calendar” (P4).  “I wish the app had reminders, like notifications in the morning to remind me to log.” (P4)  “It felt like some of the criteria in the app were too vague. I wasn’t always sure what I was supposed to do.” (P3)  “Send alerts when weekly alcohol limits are exceeded” (P2).  “Make sure that diary entries are saved and reflected in the app immediately (P8)  “Show averages of past drinking and survey responses.” (P11)  “It would be nice to see trends over time, like a graph showing my progress.” (P9) |
|  | Content suggestions | “Provide guidelines on heavy drinking to help control intake” (P8)  “It would be great if I could log more details, like my emotions or cravings. Just using a simple scale didn’t capture everything.” (P12)  “Recording drinking frequency and amount raised awareness of weekly limits.” (P15)  “There wasn’t much information about the long-term effects of drinking. Adding that would help people understand why they need to cut back.” (P17) |
|  | Other | “Integrate app with health apps for sleep records and analysis.” (P10) |
| **Clinicians** | Behavioral Impact | “By recording their mood and sleep daily while abstaining from alcohol, participants seemed to independently recognize the positive changes in their mind and body, which, in turn, reinforced their commitment to staying alcohol-free.” (C1) |
|  | Usability | “Allowing for participants to set personalized goals every week, and adding features that allow participants to record their achievement would be beneficial.” (C1)  “Features that connect clinicians and patients, like messaging or feedback options, would make follow-ups easier and more effective.” (C2)  “If patients' motivation levels for change can be assessed through the app, it will help clinicians effectively tailor their approach to patient interventions in real-life settings.” (C2) |
|  | Content | “Add a part in which participants can describe in their own words what impact alcohol had on their lives, and their personal motivation for change, such that they can go back to the messages as a reminder when the feel strong urge to drink.” (C1)  “Younger and older users have very different needs and expectations. The app should include content that’s tailored to different age groups.” (C2) |
